# Genome Sequence of the Yeast *Saprochaete ingens* CBS 517.90

**DOI:** 10.1128/MRA.01366-19

**Published:** 2019-12-12

**Authors:** Viktória Hodorová, Hana Lichancová, Stanislav Zubenko, Karolina Sienkiewicz, Sarah Mae U. Penir, Philipp Afanasyev, Dominic Boceck, Sarah Bonnin, Siras Hakobyan, Urszula Smyczynska, Erik Zhivkoplias, Maryna Zlatohurska, Eugeniusz Tralle, Alina Frolova, Leszek P. Pryszcz, Broňa Brejová, Tomáš Vinař, Jozef Nosek

**Affiliations:** a Department of Biochemistry, Faculty of Natural Sciences, Comenius University in Bratislava, Bratislava, Slovak Republic; b Kyiv Academic University, Kiev, Ukraine; c Faculty of Mathematics, Informatics and Mechanics, University of Warsaw, Warsaw, Poland; d Department of Meiosis, Max Planck Institute for Biophysical Chemistry, Göttingen, Germany; e Laboratory of Evolutionary Genomics, Vavilov Institute of General Genetics, Moscow, Russia; f Algorithms in Bioinformatics, ZBIT Center for Bioinformatics, University of Tübingen, Tübingen, Germany; g Centre for Genomic Regulation (CRG), The Barcelona Institute of Science and Technology, Barcelona, Spain; h Institute of Molecular Biology NAS RA, Yerevan, Armenia; i Department of Biostatistics and Translational Medicine, Medical University of Lodz, Lodz, Poland; j Biology Education Centre, Uppsala University, Uppsala, Sweden; k Institute of Microbiology and Virology, National Academy of Science of Ukraine, Kiev, Ukraine; l International Institute of Molecular and Cell Biology in Warsaw, Warsaw, Poland; m Institute of Molecular Biology and Genetics, National Academy of Sciences of Ukraine, Kiev, Ukraine; n Department of Computer Science, Faculty of Mathematics, Physics and Informatics, Comenius University in Bratislava, Bratislava, Slovak Republic; o Department of Applied Informatics, Faculty of Mathematics, Physics and Informatics, Comenius University in Bratislava, Bratislava, Slovak Republic; Broad Institute

## Abstract

Chromosome-scale genome assembly of the yeast *Saprochaete ingens* CBS 517.90 was determined by a combination of technologies producing short (HiSeq X; Illumina) and long (MinION; Oxford Nanopore Technologies) reads. The 21.2-Mbp genome sequence has a GC content of 36.9% and codes for 6,475 predicted proteins.

## ANNOUNCEMENT

The yeast Saprochaete ingens was originally described as Candida ingens (1) and later classified into the Magnusiomyces*/*Saprochaete clade (Dipodascaceae, Saccharomycotina, Ascomycota). In this clade, teleomorphic and anamorphic stages were named *Magnusiomyces* and *Saprochaete*, respectively. To investigate claims that *Saprochaete ingens* and Magnusiomyces ingens do not represent different reproductive stages of the same species but rather distinct taxa (2–4), we sequenced the genome of S. ingens ex-holotype strain CBS 517.90, isolated from a wine cellar in Western Cape Province, South Africa (1), and compared it to the recently determined M. ingens genome (5).

The yeasts were grown overnight in yeast extract-peptone-dextrose (YPD) medium (1% [wt/vol] yeast extract, 2% [wt/vol] peptone, and 1% [wt/vol] glucose) at 28°C, and the genomic DNA was purified using a Genomic-tip 100/G (Qiagen) (6). A total of 111,042 long reads (mean, 13,586.5 nucleotides [nt]; median, 5,776 nt; longest read, 192,848 nt) totaling 1.5 Gbp (∼71× coverage) were obtained with a MinION Mk-1B device on an R9.4.1 flow cell, using ligation kit SQK-LSK109, and base called by ONT Albacore (v. 2.3.1). A paired-end (2 × 151-nt) TruSeq PCR-free DNA library was sequenced on a HiSeq X Ten platform by Macrogen Korea, yielding 172,059,934 reads (25.98 Gbp; ∼1,226× coverage). No additional read trimming or filtering was performed. Unless otherwise noted, all tools were used with default parameters.

Eleven contigs of the initial long-read assembly (miniasm v. 0.3-r179 [7]; minimap2 v. 2.13-r852 [option -x ava-ont] [8]; polished by Racon v. 1.3.1 [option –include-unpolished] [9]) were compared with long-read assemblies by wtdgb2 v. 2.3 (options -g 20 m -x ont) (10) and Canu v. 1.7 (options genomeSize = 25m overlapper=mhap utgReAlign=true) (11). Based on the comparison, four pairs of contigs were connected, two contigs were extended to telomeres, and seven local misassemblies were corrected. A short contig containing only ribosomal DNA (rDNA) repeats was discarded, with and additional eight copies of rDNA present in contig 4. The resulting assembly was polished with short reads (four iterations of pilon v. 1.21 [12]; BWA-MEM v. 0.7.17-r1188 [option -M] [13]). The rDNA repeat and the mitochondrial genome were polished separately from the rest of the genome to avoid ambiguous alignments.

The assembly is 21.2 Mbp long and consists of five nuclear contigs (between 2.7 and 5.7 Mbp) and a mitochondrial genome (35.5 kbp). Nine nuclear contig ends are terminated by telomeric repeats (CA_3_G_5–8_)_n_, indicating five chromosomes with one telomeric region missing from the assembly. Genes were annotated using Augustus v. 3.2.3 (option –uniqueGeneId=true) (14), with initial parameters estimated from Magnusiomyces capitatus (5) and then trained on the 3,341 predicted *S. ingens* genes with at least 80% protein-level identity to their closest *M. ingens* ortholog. A total of 14 predictions were discarded due to in-frame stop codons, resulting in 6,475 nuclear protein-coding genes.

**FIG 1:**
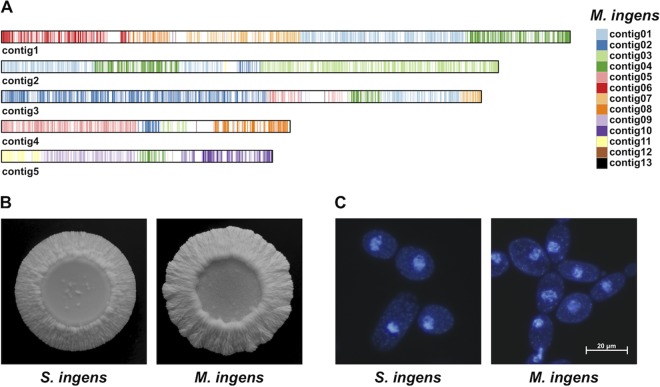
(A) Nuclear contigs of *S. ingens* CBS 517.90 colored based on alignments to contigs of *M. ingens* NRRL Y-17630 (CBS 521.90) (5). The comparison was performed using Last Aligner v. 830 (option -E1e-10) (15), postprocessed by last-split to keep only the best match at each *M. ingens* locus, and visualized using ggplot2 (16). (B) Differentiated colonies of *S. ingens* CBS 517.90 and *M. ingens* NRRL Y-17630 grown on yeast extract-malt extract-peptone (YM) plates (0.3% [wt/vol] yeast extract, 0.3% [wt/vol] malt extract, and 0.5% [wt/vol] peptone) containing 1% (wt/vol) glucose at 28°C for about 2 weeks. (C) Nuclear and mitochondrial DNA of *S. ingens* CBS 517.90 and *M. ingens* NRRL Y-17630 cells stained with 4′,6-diamidino-2-phenylindole (DAPI) and visualized using an Olympus BX50 microscope.

The nuclear genome comparison of *S. ingens* and *M. ingens* (Fig. 1A) shows that, although the genomes exhibit a long-range synteny, the alignments are fragmented and have only about 77% identity (median among alignments with at least 1,000 matches). The comparison thus demonstrates that, despite these two yeasts exhibiting many common features, such as similar assimilation profiles (3, 4) and colony and cell morphologies (Fig. 1B and C), they represent different species.

### Data availability.

The assembly has been deposited in ENA (accession no. CABVLU010000000). Illumina and MinION reads have been deposited under accession no. ERR3510534 and ERR3509916, respectively. The assembly and its annotation can also be viewed interactively in a genome browser available at http://genome.compbio.fmph.uniba.sk/.
